# Eco-Friendly Hydrogels Loading Polyphenols-Composed Biomimetic Micelles for Topical Administration of Resveratrol and Rutin

**DOI:** 10.3390/biomimetics10010008

**Published:** 2024-12-27

**Authors:** Beatriz N. Guedes, Tatiana Andreani, M. Beatriz P. P. Oliveira, Faezeh Fathi, Eliana B. Souto

**Affiliations:** 1 Laboratory of Pharmaceutical Technology, Faculty of Pharmacy, University of Porto, 4050-313 Porto, Portugal; beatriznibra08@gmail.com; 2 GreenUPorto-Sustainable Agrifood Production Research Centre, INOV4AGRO, Biology Department, Faculty of Sciences of University of Porto, Rua do Campo Alegre s/n, 4169-007 Porto, Portugal; tatiana.andreani@fc.up.pt; 3 Chemistry Research Center (CIQUP), Institute of Molecular Sciences (IMS), Department of Chemistry and Biochemistry, Faculty of Sciences of the University of Porto (FCUP), Rua do Campo Alegre 687, 4169-007 Porto, Portugal; 4 REQUIMTE/LAQV, Department of Chemical Sciences, Faculty of Pharmacy, University of Porto, Rua Jorge Viterbo Ferreira, 280, 4050-313 Porto, Portugal; beatoliv@ff.up.pt; 5 UCD School of Chemical and Bioprocess Engineering, University College Dublin, Belfield, D04 V1W8 Dublin, Ireland

**Keywords:** biomimetic micelles, hydrogel, olive pomace, polyphenols, resveratrol, rutin

## Abstract

In this study, we describe the development of hydrogel formulations composed of micelles loading two natural antioxidants—resveratrol and rutin—and the evaluation of the effect of a by-product on the rheological and textural properties of the developed semi-solids. This approach aims to associate the advantages of hydrogels for topical administration of drugs and of lipid micelles that mimic skin composition for the delivery of poorly water-soluble compounds in combination therapy. Biomimetic micelles composed of L-α-phosphatidylcholine loaded with two distinct polyphenols (one non-flavonoid and one flavonoid) were produced using hot shear homogenisation followed by the ultrasonication method. All developed micelles were dispersed in a carbomer 940-based hydrogel to obtain three distinct semi-solid formulations, which were then characterised by analysing the thermal, rheological and textural properties. Olive pomace-based hydrogels were also produced to contain the same micelles as an alternative to respond to the needs of zero waste and circular economy. The thermograms showed no changes in the typical profiles of micelles when loaded into the hydrogels. The rheological analysis confirmed that the produced hydrogels achieved the ideal properties of a semi-solid product for topical administration. The viscosity values of the hydrogels loaded with olive pomace (hydrogels A) proved to be lower than the hydrogels without olive pomace (hydrogels B), with this ingredient having a considerable effect in reducing the viscosity of the final formulation, yet without compromising the firmness and cohesiveness of the gels. The texture analysis of both hydrogels A and B also exhibited the typical behaviour expected of a semi-solid system.

## 1. Introduction

Hydrogels are a class of three-dimensional (3D) polymer networks with high water retention capacity [1]. They can be composed of any hydrophilic polymer obtained by chemical or physical cross-linking [2]. Hydrogels have a wide variety of physical properties and chemical compositions that can be tailored to a specific need, thus showing numerous applications, from studies of physiological and pathological mechanisms to tissue regeneration and drug delivery.

Hydrogels have certain properties that make them highly suitable as drug delivery vehicles, such as being biodegradable and biocompatible, having the ability to carry different drugs, maintaining a high concentration of active ingredients for a long period in the site of interest and modifying the release profile of loaded drugs [3,4].

Hydrogels are being increasingly used to develop dressings for wound healing as an alternative to more traditional treatments, such as bandages and gauzes. Hydrogels have several beneficial properties for treating skin wounds since they are compatible with biological tissues, have the ability to retain a high amount of water due to their high porosity, and can simultaneously keep the wound moist and absorb the exudates [5,6]. Since they are non-irritating, do not react with biological tissue and are permeable to metabolites, hydrogels are appropriate for all stages of the skin healing process, namely, haemostasis, inflammation, cell migration/proliferation and maturation [7]. Thus, hydrogel dressings are being widely studied for accelerated wound healing [8,9,10,11]. To expedite wound treatment, a large variety of hydrogel dressings can be applied to several types of skin wounds, depending on the need. This resulted in the rapid introduction of a range of dressing materials into the pharmaceutical market, such as sheets, saturated gauze, and gels [12]. With the development of nanotechnology, nanoparticles loaded with bioactives of interest are being combined with other biomaterials to create innovative hybrid systems for new applications [13,14,15]. Following this evolution, the addition of nanoparticles to hydrogels is gaining even more interest [6,16,17].

Before a bioactive ingredient is able to show its health-beneficial effects, it undergoes a variety of processes before reaching its target [18]. It needs to become bioavailable and bioaccessible, attributes that are mostly affected by the biofunctional properties of the bioactive compounds [19]. Regarding skin delivery of antioxidants, such as resveratrol and rutin, it is expected that they go deeper into the skin layers yet without reaching the systemic circulation. The degree of permeation of the skin and controlled penetration of bioactive ingredients for dermopharmaceutical use can be governed by the use of specific delivery systems. Among these, lipid delivery systems play a relevant role because they act as permeation enhancers. Lipid micelles are a typical example of successful delivery systems selected to improve the solubility of poorly water-soluble compounds for that specific purpose, besides allowing the modification of the release profile of drugs and targeting them to specific locations, and thus reducing the risk of side effects from systemic distribution of drugs [20,21].

**Figure 1:**
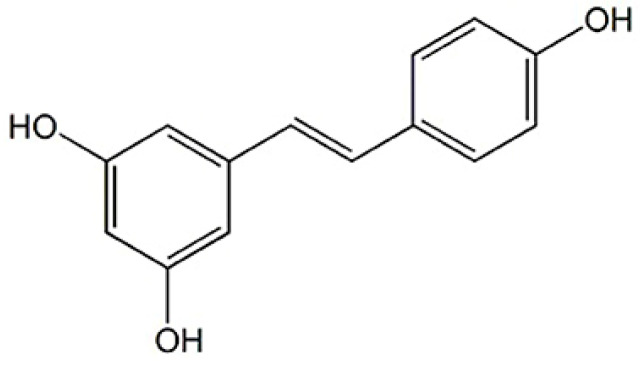
Chemical structure of Resveratrol (reproduced after Chedea, Veronica Sanda et al. (2021) [27], under the terms and conditions of the Creative Commons Attribution (CC BY) license.

**Figure 2:**
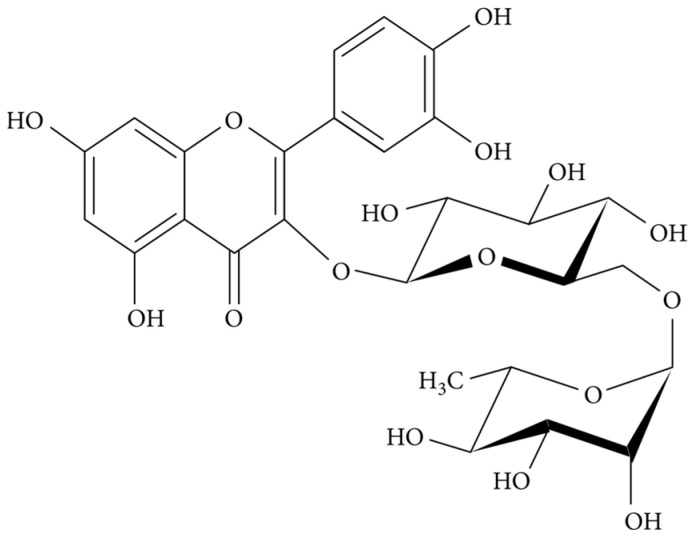
Chemical structure of rutin (reproduced after Enogieru, Adaze Bijou et al. (2018) [28], under the terms and conditions of the Creative Commons Attribution (CC BY) license.

In this work, we describe the development of semi-solid formulations using previously produced biomimetic micelles containing resveratrol (Figure 1) and rutin (Figure 2) alone and in combination in the same micelle (dual loading). Micelles based on L-α-phosphatidylcholine (as the core ingredient) mimic the physiology of cell membranes and are considered biocompatible for skin drug delivery, acting as modulators of skin permeation [22]. These biomimetic micelles were selected for the loading of resveratrol and rutin to improve their solubility profile and enhance their dermal delivery. These two bioactives are obtained from natural sources, namely plants and fruits, and both offer interesting activities of pharmacological and cosmetical interest. Their formulation into biomimetic micelles is aimed at overcoming the poor water solubility, poor chemical stability and low skin permeability associated with resveratrol and rutin [23,24,25,26].

*Olea europaea* L., olive tree, is one of the oldest cultivated trees in the Mediterranean region, and it is native to Asia Minor and Syria [29]. In the Mediterranean diet, olive tree products, such as olive oil and olives, are widely consumed [30,31]. However, the olive oil industry is associated with environmental issues due to the high waste production [32]. The wastes that generate the greatest ecological concern due to high organic toxicity and low pH include olive pomace, olive leaves and olive mill wastewater [33]. Olive pomace is a semi-solid residue with a high amount of water in its constitution (60%) and has low pH. Olive pomace also contains a high content of phenolic compounds and retains most of the phenolic content of the olive [34,35]. Only 1–2% of the phenolic content is present in olive oil [34,36]. However, as a by-product, olive pomace becomes phytotoxic and non-biodegradable [29,37], but it is of great interest to several industries due to its varied bioactivities and health-promoting properties, and new forms of reuse are being proposed.

It has been demonstrated that olive pomace, as well as its bioactive phenolic compounds, show anti-inflammatory [38,39,40], antioxidant [41,42,43] and photoprotective activities [42,44,45,46]. Furthermore, its application has also been reported in cosmetics and dermatological products for the treatment of skin diseases using different delivery systems [47,48]. Hydrogels based on olive pomace are one example of these delivery systems. Therefore, to value these olive by-products, their sustainable recovery has been highlighted in several research studies [33]. Resveratrol and rutin are non-toxic phytochemicals with several biological properties of interest to the pharmaceutical and cosmetic industries. However, the low stability and low water solubility of these compounds negatively affect their bioavailability and, in the case of skin, their permeability, which limits their applications. This work aimed to develop techniques that would enable greater stability in the encapsulation of resveratrol and rutin, together with the reuse of the by-product olive pomace, to obtain an innovative dermocosmetic product capable of effective delivery of these bioactives with appealing properties to the consumer, such as smoothness and eco-friendly character.

## 2. Materials and Methods

### 2.1. Materials

The materials used in the study included Polysorbate 80 (Acofarma, Barcelona, Spain), Sorbitan monostearate 80 (Acofarma, Barcelona, Spain), L-α-Phosphatidylcholine (Sigma-Aldrich, St. Louis, MO, USA), Resveratrol (Fagron, Barcelona, Spain), Rutin (Acros Organics, Geel, Belgium), Carbomer 940 (Fagron, Barcelona, Spain), Sodium Hydroxide (Sigma-Aldrich, St. Louis, MO, USA), Methylparaben (Acofarma, Barcelona, Spain), Propylparaben (Acofarma, Barcelona, Spain) and Propylene glycol (Acofarma, Barcelona, Spain). Olive pomace was obtained from local producers. Deionised water (Milli-Q water home supplied) was used throughout all experiments.

### 2.2. Production of Resveratrol- and Rutin-Loaded Biomimetic Micelles

**Figure 3:**
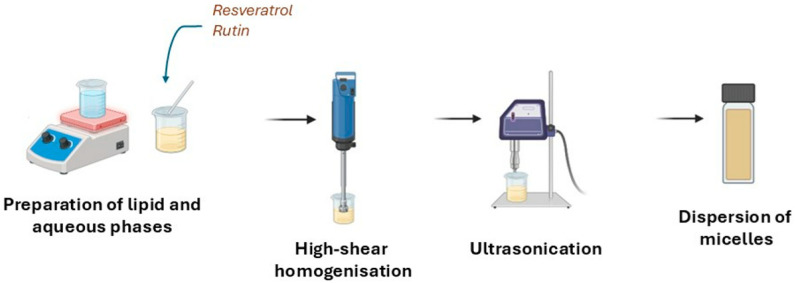
Schematic representation of the production of resveratrol- and rutin-loaded micelles.

For the production of micelles, the hot shear homogenisation technique followed by the ultrasonication method was used as previously described, with adaptations [49] (Figure 3). The compositions of the different prepared micelle formulations are shown in Table 1. Briefly, for all samples (Mc1, Mc2 and Mc3), the active compound concentration was 0.1% *(w*/*w)*. This, mixed with soy lecithin (L-α-Phosphatidylcholine), which is a phospholipid, formed the lipid phase of the system. The aqueous phase was composed of non-ionic surfactants (Polysorbate 80 and/or Span 80) dissolved in ultra-purified water.

The aqueous phase was added to the lipid phase, and the obtained mixture was heated under constant stirring until boiling. The resulting dispersion was stirred at high speed (7000 rpm) using an Ultra-Turrax (IKA, Staufen, Germany) for 5 min. This mixture was subsequently subjected to sonication with a probe for 5 min and an amplitude of 70%. The obtained formulations were then stored at room temperature until further use.

### 2.3. Production of Resveratrol- and Rutin-Loaded Micelles Composed Hydrogels

For the acceptance of a topical product, there are several important parameters to be considered, such as its easy application to the skin, the sensorial properties (brightness and amount of residue) and the appearance of the product.

**Figure 4:**
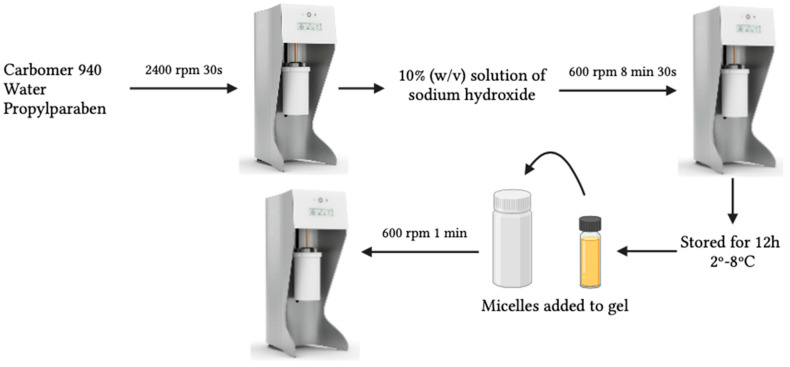
Schematic representation of the production of resveratrol- and rutin-loaded micelles composed hydrogels.

As micelle dispersions do not have an adequate consistency for application to the skin, as they are liquid systems, their transformation into semi-solid systems becomes an attractive solution for product acceptability, besides improving the stability of micelles by reducing the risk of forming aggregates over the shelf-life [50,51]. Thus, hydrogels based on micelles (Hydrogel A and Hydrogel B) were also developed (Figure 4).

Carbomer was used to improve the viscosity of the system and obtain suitable consistency levels for topical application. First, a 2% (*w*/*v*) carbomer 940 gel was prepared with the aid of the Unguator^®^ mixer (Gako Deutschland GmbH, Schesslitz, Germany). Carbomer 940, water and propylparaben were weighed and mixed at a speed of 2400 rpm for 30 s. Propylparaben was used as a preservative, and a 10% (*w*/*v*) solution of sodium hydroxide (0.1 M) was used to adjust the pH value of the carbomer up to 6.5–7. The solution of sodium hydroxide was added to the mixture dropwise, under gentle stirring of 600 rpm for 8 min and 30 s, to induce polymer gelation until reaching pH 6.5. The developed hydrogel was kept still for the following 12 h to eliminate the presence of any existing air bubbles at a controlled temperature of 2–8 °C for further studies. For the final preparation of the hydrogel loaded with micelles, the previously prepared 2% carbomer gel was used.

**Figure 5:**
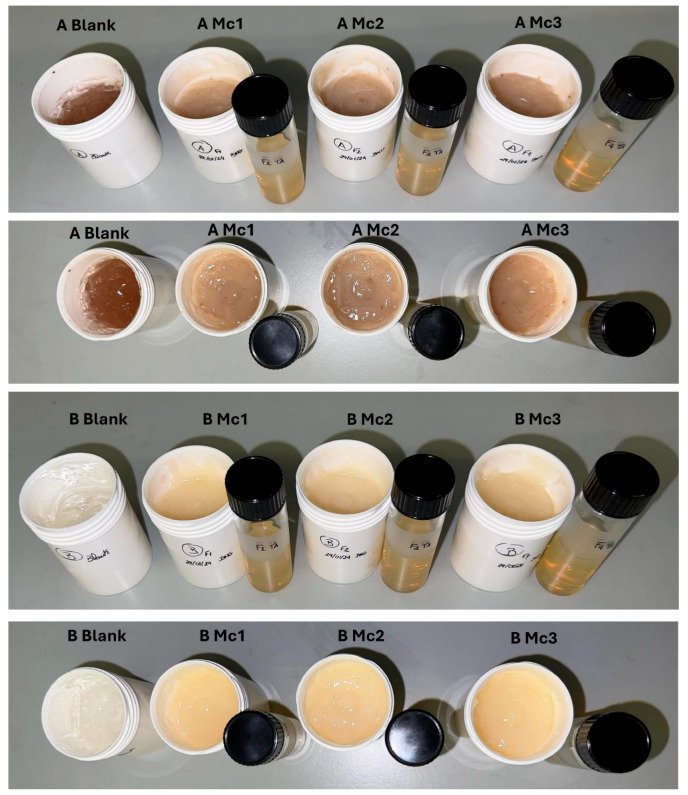
Hydrogels A (front and top) and hydrogels B (front and top).

The micelles were added to the gel and homogenised for 1 min at 600 rpm in the Unguator^®^. The final formulation consisted of 50% of 2% carbomer gel, 49.7% of micelles and 0.3% of olive pomace (hydrogel A) and 50% of 2% carbomer gel and 50% of micelles (hydrogel B). The composition of all developed hydrogel formulations is represented in Table 2 and Table 3. Figure 5 depicts the photographs of all the produced hydrogels.

### 2.4. Differential Scanning Calorimetry

Differential Scanning Calorimetry analysis was used to ascertain the physical state and thermal properties of the micelles-loaded hydrogels. This task was carried out on a DSC 200 F3 Maia^®^ (NETZSCH, Selb, Germany). The equipment consists of an oven with two different chambers. One chamber houses the crucible that contains the sample, and the other houses the reference crucible (position 0). The samples were weighed (approximately 5 mg) in an aluminium crucible that was subsequently closed. The analysis consists of a temperature program between 20 °C and 70 °C, at a heating rate of 10 °C/min, to which the samples are subjected to record the corresponding thermogram. Data analysis was performed using the thermal analysis software “Proteus^®^ 6.1.0B” software (NETZSCH, Selb, Germany).

### 2.5. Rheological Analysis

The viscoelastic properties of the developed hydrogels were evaluated using a Rheometer Kinexus Lab + (Malvern, Worcestershire, UK). The hydrogels were measured at a temperature of 25 °C with a spacing of 1 mm gap between plates. A portion of each sample under analysis was placed on the lower plate of the equipment, where a torque was subsequently applied, which promoted the shear stress. The frequency used in the analysis was from 0.1 to 10.0 Hz, in oscillation mode and 1% deformation. Data were compiled and collected using the software “rSpace for Kinexus Lab +” (version 1.75 Malvern Instruments).

### 2.6. Texture Analysis

To analyse the texture of the different samples, a texture analyser TA-XT2i© (Stable Micro Systems, Godalming, UK) and the software “Exponent” (version 6.1.12.0) were used. The sample was placed in the equipment, and a P/0.5 ½ Dia Delrin Aoacque probe penetrated it with a 5 Kg load cell. The firmness and cohesiveness were evaluated at room temperature (22–25 °C) for all samples. Data acquisition and analysis were performed using the software “Texture Expert^®^” (version 6.1.12.0).

## 3. Results and Discussion

**Figure 6:**
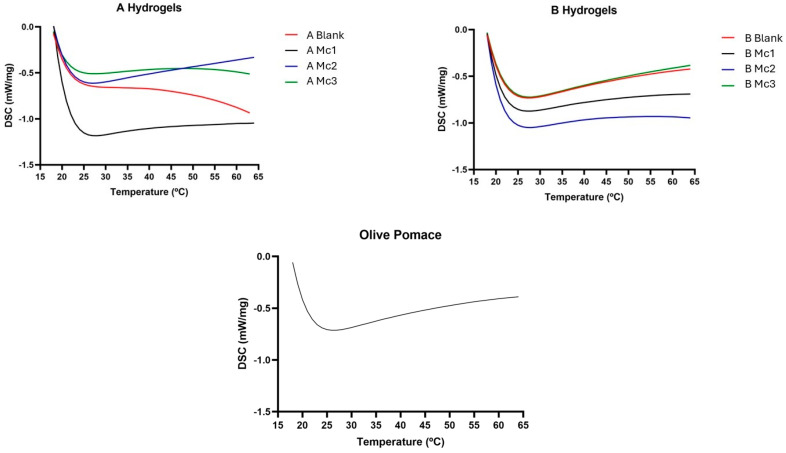
DSC analysis of the developed hydrogels and olive pomace.

DSC was carried out to analyse the thermal properties of hydrogels A and B before (blank) and after dispersing the loaded micelles, in comparison to olive pomace at the same temperature range (20–65 °C) and the results are presented in Figure 6. All samples presented similar endothermic profiles. No peaks were recorded in the analysis of the different A and B hydrogel samples, corroborating the idea that the system does not have a solid matrix.

**Figure 7:**
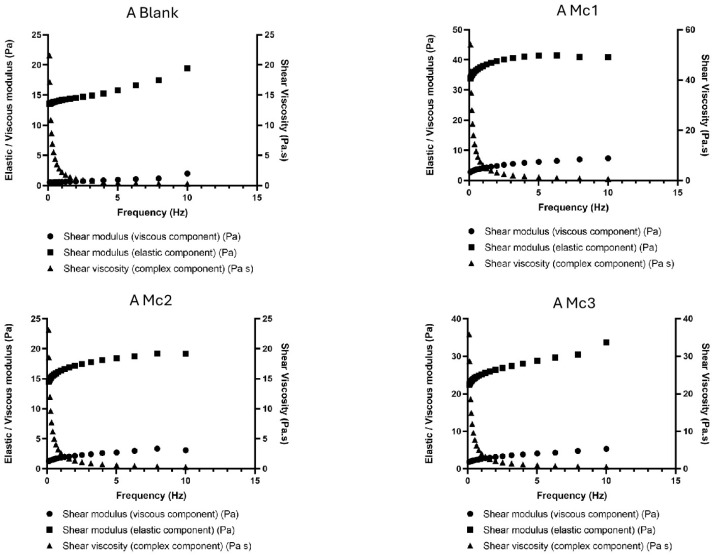
Oscillation frequency sweep test of Hydrogels A.

**Figure 8:**
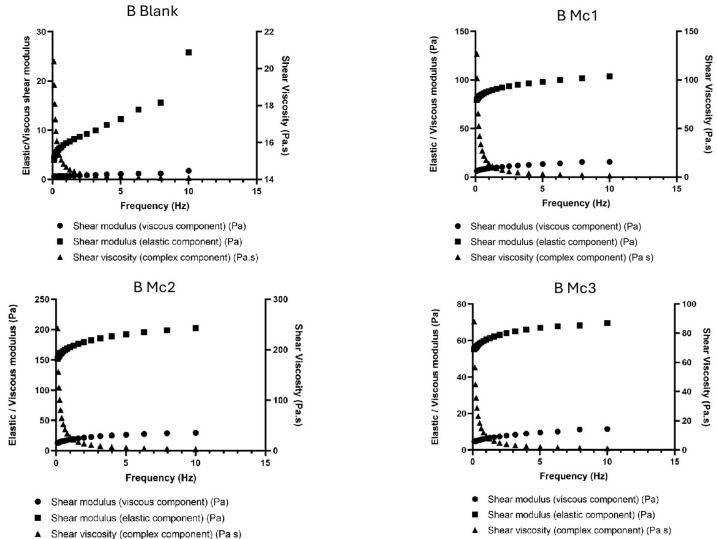
Oscillation frequency sweep test of Hydrogels B.

The developed hydrogels were all composed of 2% carbomer, which is a well-known high molecular weight, hydrophilic, crosslinked polymer of polyacrylic acid. Carbomer creates a three-dimensional polymer network that absorbs water and exhibits transitory, reversible interchain entanglements making it more versatile and robust than other chemical hydrogels [52]. Its rheological properties, whether dispersed in water or in water/glycerol mixtures, have been extensively characterised [52]. In our work, we used the polymer dissolved in water to obtain the semi-solid base for the dispersion of our biomimetic micelles. The oscillatory study (Figure 7 and Figure 8) was carried out to compare the rheological profile of the obtained micelles-containing hydrogels with and without the presence of olive pomace. This test describes the system response as a function of frequency at constant shear strain and provides information on the storage modulus (elastic component, G’), loss modulus (viscous component, G”) and shear viscosity. The G’ resembles the amount of energy that the dispersion needs to be distorted, while the G” reflects the energy lost during deformation. As the frequency range increases, the shear rate also increases, thus requiring more energy and consequently increasing G” and G’. This test is relevant to be run in the final product to assess whether it has the appropriate rheological properties to be administered on the skin. In our work, we used the polymer (2%, *m*/*v*) dissolved in water, neutralised with NaOH 0.1 M until reaching pH 6.5, in which micelles containing the bioactives were dispersed. The results show that, for all tested formulations depicted in Figure 7 and Figure 8, the G′ modulus (the storage or elastic modulus) was always much higher than the G″ modulus (the loss or viscous modulus) throughout the entire frequency range (0.1–10 Hz). These results indicate that the elastic properties dominate the viscous behaviour [53]. These data point out to the presence of a gel-like structure and indicate that the system is more elastic than viscous, which is a characteristic of viscoelastic systems, wherein the microstructure retains energy from oscillations and relaxes adequately to discharge a portion of that energy through microstructural rearrangements [53]. When a viscoelastic material experiences stress, its response consists of elastic deformation (which stores energy) and viscous flow (which loses energy), confirming the suitability of the developed semi-solids for topical administration.

It can also be observed that the G′ and G″ of all samples are strongly dependent on the frequency, indicating short relaxation times for the microstructures within the applied frequency range. Viscosity was also found to be frequency-dependent as its values decreased significantly with increasing frequency, particularly when starting the test. This behaviour is typical of viscoelastic semi-solids and can be found for standard topical dosage forms indicating that the formulations can be easily rubbed onto the skin [54]. When comparing blank hydrogels with those containing drug-loaded micelles, the presence of these latter increased the viscosity of the semi-solids in general terms but did not compromise the firmness and cohesiveness of the hydrogels (Table 4). The viscosity of the hydrogels A and B was also strongly dependent on the presence of olive pomace. In hydrogel A (Figure 7), containing olive pomace, the viscosity is considerably lower in all samples (A Mc1, A Mc2 and A Mc3) compared to hydrogel B formulations (Figure 8), showing that olive pomace reduces the viscosity of the systems [55]. We could also see that, in hydrogels B, a greater distance was recorded between the moduli G’ and G” for each formulation compared to hydrogels A, showing a greater capacity to retain energy (storage modulus) than to lose energy (loss modulus).

Texture can be understood as the physical characteristics perceived through touch, and it is related to the deformation caused by a force. These characteristics are analysed and measured through distance, force and time. Through texture analysis, we can obtain the necessary knowledge about the structural properties of the products to predict the behaviour of the formulation in vivo [56]. The texture analysis of topical pharmaceutical formulations is an important assay because the acceptability of the product by the customer/patient will depend mainly on its application and organoleptic attributes. Texture analysis was carried out by compressing the developed samples, with a specific probe, at a constant speed. As a result, a profile is generated in which the positive part represents the force necessary to penetrate the sample, while the negative part refers to the force necessary for the probe to be removed from the sample and return to its initial position. The maximum force recorded represents firmness, and the negative force peak of the profile shows cohesiveness. Firmness measures the force required to produce deformation in the gel and measures the ability of the gel to resist clearance in the target area. Cohesiveness measures the internal binding strength of the final hydrogel structure [57,58]. The results obtained from the texture analysis of different samples are presented in Table 4. Comparing the blank hydrogels with the hydrogels containing bioactive-loaded micelles, these latter registered lower firmness values, i.e., the addition of micelles reduced the firmness of blank hydrogels (A Blank 0.3305 N and B Blank 0.3049 N). The first positive region that is recorded in the texture analysis is related to firmness, and higher values mean a firmer gel. The two lowest firmness values are found in hydrogel A, in samples A Mc2 and A Mc3, 0.2470 N and 0.2323 N, respectively.

The first negative force that is recorded in the texture analysis is related to the cohesiveness of the gel. The sample A Mc3, the one based on the dual loaded-micelles-composed hydrogels containing olive pomace, was found to be the least cohesive hydrogel with the force closest to zero (0.1302 N). In general, hydrogels B were found to be more cohesive than hydrogels A, except for sample B Mc1.

In summary, our results indicate that dispersing micelles into hydrogels may improve both rheological properties and the texture of semi-solid hydrogels, whereas the presence of that olive pomace does not compromise the firmness and cohesiveness of the systems as they were recorded within the same range of values. This by-product can further be exploited for its emollient, moisturiser and nourisher properties [59]. These findings highlight the interest in the use of olive pomace as a new ingredient of hydrogel formulations for skin administration.

## 4. Conclusions

The aim of this study was to prepare a new eco-friendly hydrogel loaded with different natural antioxidant compounds, with the reuse of a by-product (olive pomace). Hydrogels containing resveratrol-loaded micelles, rutin-loaded micelles, or resveratrol–rutin-loaded micelles were successfully developed with and without olive pomace. The hydrogels presented G’ values much higher than the G” values across the whole frequency range, indicating a gel-like structure and showing that the systems were more elastic than viscous, confirming this key attribute for topical administration. It was also found that the viscosity of hydrogels A and B was strongly dependent on the presence of micelles and olive pomace; while the presence of micelles increased the viscosity of the gels, when adding olive pomace, the system showed lower viscosity yet without compromising firmness and cohesiveness. From the texture analysis, hydrogels containing olive pomace have a more rigid, firm and cohesive matrix than those hydrogels based only on carbomer. The results of this analysis agree with those of the rheological analysis and corroborate the interest in using by-products in pharmaceutical and cosmetic formulations.

## 5. Patents

The data described in this manuscript are part of the invention that led to the submission of a patent application (NPAT554—NanoMICDermo: Olive pomace-based hydrogels containing Novel Micelles structure loaded with rutin and resveratrol for topical use) (Nr. 119567, 28 June 2024).

## Figures and Tables

**Table 1:** Composition of resveratrol- and rutin-loaded biomimetic micelles.

Sample	Resveratrol (%, *w*/*w*)	Rutin (%, *w*/*w*)	Soy Lecithin (%, *w*/*w*)	Polysorbate 80 (%, *w*/*w*)	Span 80 (%, *w*/*w*)	Water ad. (%, *w*/*w*)
Mc1	0.10	-	4.90	1.00	-	100
Mc2	-	0.10	4.90	0.60	0.400	100
Mc3	0.05	0.05	4.90	1.00	-	100

**Table 2:** Composition of 100 g of 2% carbomer 940 gel.

Carbomer 940 (g)	10% Sodium Hydroxide 0.1 M (mL; *w*/*v*)	Propylparaben (g)	Purified Water ad (g)
2	3.2	1.5	100

**Table 3:** Composition of hydrogels A and hydrogels B.

Materials	A Blank Hydrogel	A Mc1	A Mc2	A Mc3	B Blank Hydrogel	B Mc1	B Mc2	B Mc3
2% Carbomer 940 (g)	29.85	15	15	15	30	15	15	15
Olive Pomace (g)	0.15	0.15	0.15	0.15	-	-	-	-
Resveratrol-loaded micelles (g)	-	14.85	-	-	-	15	-	-
Rutin-loaded micelles (g)		-	14.85	-	-	-	15	-
Resveratrol + Rutin-loaded micelles (g)	-	-	-	14.85	-	-	-	15

**Table 4:** Textural properties of the hydrogels.

Parameters	A Blank	A Mc1	A Mc2	A Mc3	B Blank	B Mc1	B Mc2	B Mc3
Firmness (N)	0.3305	0.2628	0.2470	0.2323	0.3049	0.2592	0.2745	0.2886
Cohesiveness (N)	−0.1328	−0.1536	−0.1391	−0.1302	−0.1427	−0.1416	−0.1538	−0.1341

## Data Availability

Data will be made available upon reasonable request.
